# Sleep Bruxism in Children—What Can Be Learned from Anamnestic Information

**DOI:** 10.3390/jcm12072564

**Published:** 2023-03-29

**Authors:** Alona Emodi-Perlman, Yarden Shreiber-Fridman, Shani Kaminsky-Kurtz, Ilana Eli, Sigalit Blumer

**Affiliations:** 1 Department of Oral Rehabilitation, The Maurice and Gabriela Goldschleger School of Dental Medicine, Sackler Faculty of Medicine, Tel Aviv University, Tel Aviv 6139001, Israel; 2 The Maurice and Gabriela Goldschleger School of Dental Medicine, Sackler Faculty of Medicine, Tel Aviv University, Tel Aviv 6139001, Israel; 3 Department of Pediatric Dentistry, The Maurice and Gabriela Goldschleger School of Dental Medicine, Sackler Faculty of Medicine, Tel Aviv University, Tel Aviv 6139001, Israel

**Keywords:** sleep bruxism, children, anamnesis, ear tubes, tooth wear, Eustachian tubes

## Abstract

Sleep bruxism (SB) is a masticatory muscle activity during sleep, and its clinical manifestation in young children is still unclear. The aim of the present study was to evaluate the role of anamnestic information in predicting possible SB in children aged 4–12 years. In a cross-sectional retrospective exploratory study, the dental files of 521 children were examined with regard to the following anamnestic information: gender, age, medical conditions associated with ear, nose, and throat (ENT), respiratory disorders, use of methylphenidate (Ritalin), oral habits, and bruxing during sleep. A child was defined as presenting possible SB when a positive report was received from parents regarding such behavior (SB positive, No. = 84). There were no age- and/or gender-wise differences between SB-positive children and children whose parents did not report SB behavior (SB negative). SB-positive children suffered more from ENT and respiratory disorders than children without SB. Additionally, the use of pacifiers/finger sucking, as well as snoring, were more common among SB-positive children as compared to their SB-negative counterparts (Chi-square). The variables which were found to significantly increase the odds of possible SB in children were mouth breathing, ENT problems, and use of a pacifier or finger sucking (forward stepwise logistic regression). Clinicians should look for clinical signs of possible SB in children whose anamnesis reveals one or more of these anamnestic signals.

## 1. Introduction

Bruxism is a repetitive jaw muscle activity characterized by the clenching or grinding of teeth and/or bracing or thrusting of the mandible [1]. It refers to two distinct masticatory muscle behaviors according to their circadian manifestation: awake bruxism (AB) and sleep bruxism (SB) [2]. SB is a masticatory muscle activity during sleep that is characterized as rhythmic (phasic) or non-rhythmic (tonic) [3]. SB etiology is multifactorial and involves biological, psychological, and genetic factors [4,5,6,7,8,9,10]. 

Most of the studies carried out on SB refer to adults, as do the proposed SB grading systems. In 2018, an international consensus group proposed a three-grade grading system for bruxism: (i) Possible sleep/awake bruxism based on a positive self-report only; (ii) Probable sleep/awake bruxism based on a positive clinical inspection, with or without a positive self-report; and (ii) Definite sleep/awake bruxism based on a positive instrumental assessment, with or without a positive self-report and/or positive clinical signs [3]. 

While the gold standard for definite SB diagnosis remains instrumental evaluation, the high expense and intricacy involved with it leave possible and probable SB as the most assessed bruxism grades in cross-sectional population studies [3,11,12]. Sending children to a sleep laboratory is even more complicated than an instrumental assessment of adults and results in low parental compliance [12]. For example, the gold standard for the diagnosis of obstructive sleep apnea syndrome (OSAS) is polysomnography (PSG). However, due to the problems in performing PSG in children, alternative diagnostic methods, such as sleep clinical records and nocturnal oximetry testing, have been suggested to diagnose OSAS [13]. 

Information achieved through a clinical dental examination can also be problematic in a young child. In mixed dentition, tooth wear is usually a combination of abrasion, attrition, and erosion [14]. Clinically assessed tooth wear is more intense in the primary dentition, as the primary teeth exhibit a lower degree of mineralization than permanent teeth [15]. Considering that in children tooth wear increases with age until reaching the end of primary dentition at a prevalence of 80% [16,17], the assessment of clinical signs of SB in young children is more complex than in adults. 

Assessment of SB in children cannot rely on self-report and should rather consider parental reports regarding their child’s behavior during sleep. Parental information was shown to be an effective method for the detection of behavioral and developmental problems [15,18]. Moreover, parental reports of sleep disorders were found to be consistent with objective measurements [19,20]. Thus, possible SB, which relies on parental reports, remains the most feasible grade of SB evaluation in young children.

Several clinical features have been associated with SB in children, such as sleeping position [21], sleep-related breathing disorders (SRBD), and snoring [22,23,24]. Some suggest that the masticatory muscle activity during SB acts as a protective factor in order to open an obstructed airway in SRBD. Another plausible explanation is that oral breathing expressed in snoring interferes with the sleep cycle and affects brain oxidation, leading to painful and involuntary muscle contraction of the facial muscles, causing SB [1,3,25]. To date, the full nature of associations between bruxism and additional anamnestic and clinical findings among children is still unclear. 

The aim of the present study was to evaluate the role of anamnestic data as initial signals for the presence of possible SB in young children.

## 2. Materials and Methods

In a retrospective cross-sectional exploratory study, the medical files of children aged 4–12 years who arrived for treatment at the Department of Pediatric Dentistry, School of Dental Medicine, Tel Aviv University between the 2015 and 2023, were examined. Informed consent to participate in this study was given by the children’s parents (or legal guardians). The age of 4 years was selected as the youngest age for inclusion because it is the habitual age for the diagnosis and treatment of enlarged tonsil problems [26,27]. The age of 12 was selected as the oldest age for inclusion because it is considered the average age for starting orthodontic treatment.

Exclusion criteria were as follows: children with abnormalities and/or systemic diseases that may affect the results of this study (e.g., Autistic Spectrum Disorder, mental retardation, cerebral palsy), and children with present active orthodontic equipment or who underwent orthodontic treatment in the past.

This study received the approval of the ethics committee of Tel-Aviv University (No 0001116-3).

### 2.1. Pilot Analyses

To date, there are no conclusive data regarding which anamnestic parameters can serve as initial signs of SB in young children. Therefore, a pilot study was carried out to determine the variables to be included in the final study. 

In the pilot study, the medical files of 263 children aged 4–12 years who had full anamnestic records were examined. The anamnestic questionnaire was based on pediatric medical history by the American Academy of Pediatric Dentistry [28] and referred (among others) to gender, age, former hospitalizations/surgeries, use of medications, diagnosed diseases and/or disorders, oral habits, and parental report regarding child’s bruxing during sleep. 

Results of the pilot analyses revealed significant differences between children with SB and children without SB (see paragraph 2.3, below), with regard to the following parameters: insertion of ear tubes (8.9% versus 2.8%, respectively), presence of respiratory disorders (13.3% versus 4.1%, respectively), an oral habit of mouth breathing (31.1% versus 15.6%, respectively), and an oral habit of tongue pushing (24.4% versus 11.5%, respectively). 

### 2.2. Final Study

The results of the pilot analysis served as indicators concerning the parameters to be included in the final study. A recent meta-analysis showed that children and adolescents with a definitive diagnosis of ADHD are a greater chance of developing sleep and awake bruxism than those without this disorder [29]. Additionally, findings of a former study by the present study group showed that the use of methylphenidate (Ritalin) significantly increased the odds of sleep-related breathing disorders in children (in addition to other variables such as mouth breathing and snoring) [22]. Therefore, the use of Ritalin was added to the anamnestic parameters pointed out by the pilot analysis. 

Based on the above, the following anamnestic parameters were extracted from subjects’ files:

1. Demographic variables—gender, age
2. Has the child been treated for Ear, Nose, and Throat (ENT) disorders—periodic fluid drain from the middle ear, application of ear tubes.
3. Has the child undergone a Tonsillectomy.
4. Has the child been suffering from diagnosed respiratory disorders: shortness of breath, asthma, croups, ear nose or throat polyps, bronchiolitis, bronchitis, stridor, or dyspnea.
5. Does the child use Methylphenidate (Ritalin)
6. Performance of oral habits—night drinking, finger sucking, use of a pacifier, nail biting, mouth breathing, tongue thrusting, snoring at night. All questions referred to the past and/or present.
7. Parental report regarding the child’s bruxing during sleep, past and/or present.

### 2.3. Definition of Possible SB

The SB grading systems, as suggested by the international consensus group, refer to a positive self-report of an adult subject or report of his/her sleeping partner regarding bruxing activity during sleep. Regretfully, young children’s self-report concerning SB is not reliable and diagnosis of SB in children is mostly based on reports of family members that describe the characteristic sounds of teeth grinding during sleep [30]. The use of a single question posed to parents/caregivers regarding the possible presence of SB via the existence of dental tightening or audible noises related to teeth grinding is an accepted way to determine SB in children [31]. Despite its limitations, parental reports of SB remain important for epidemiological studies with large samples [32]. Therefore, in the present study, the definition of possible SB was based on the parental report regarding the child’s bruxing during sleep in the past and/or present. 

### 2.4. Data Analysis

Data were analyzed using SPSS software (IBM SPSS statistics 27.0, Armonk, NY, USA). Chi-square and *t*-test analyses were used to compare between the groups with regard to the collected data. 

In a second step, a forward stepwise logistic regression was used to determine which of the variables could predict the presence of SB in children (SB positive).

## 3. Results

### 3.1. Population

Dental files of 1911 children were examined; 901 files were excluded due to incomplete anamnestic records; 416 children were excluded due to age not within the definition of this study parameters; and 73 children were excluded due to orthodontic treatment (present or past) or due to medical conditions as defined in Materials and Methods. 

Final analyses referred to 521 children aged 4–12 years (mean age 6.75 ± 2.40, 49.1% female). Children participating in the pilot study were part of the final sample. 

Of the 521 participating children, 16.1% were defined as suffering from possible SB according to their parents’ reports. No age and/or gender differences could be detected between children defined as presenting possible SB (SB positive, No. = 84) and children with no SB (SB negative, No. = 437). Significant differences between SB-positive and SB-negative groups could be observed regarding ENT problems, respiratory disorders, finger sucking or use of a pacifier, mouth breathing, and snoring (Table 1).

### 3.2. Multivariate Analysis

In an effort to determine which of the variables increases the odds of possible SB in children (SB positive), a forward stepwise logistic regression was used. The variables entered into the equation were those in which significant differences between SB-positive and SB-negative groups were found. Variables that were found to significantly increase the odds of possible SB in children were mouth breathing, ENT problems (periodical fluid drainage/insertion of ear tubes), and use of a pacifier or finger sucking (in the past or present). (Table 2)

## 4. Discussion

The gold standard for the assessment of SB is instrumental approaches such as EMG recordings of sleep muscle activity. Non-instrumental approaches are mostly based on self-report (e.g., questionnaires, diaries). Neither of these approaches is feasible for young children. Additional information can be gathered by dental-related signs of events potentially due to bruxism, such as mechanical tooth wear [3]; however, this can also be problematic in primary and mixed dentition [15,16,17]. 

The literature on assessing SB in children is controversial. While some studies show a positive association between parental reports and tooth wear [14,33,34,35,36], others do not support this association. Restrepo et al. showed that parental reports of their children’s sleep tooth grinding are not necessarily associated with tooth wear in mixed dentition [37]. In a recent summary, Manfredini et al. [12] claim that parental report of tooth grinding remains the most diffused option for performing studies on a large scale. 

The prevalence of Possible SB in children in the present study was about 16%, which is on the lower scale of the data presented in a recent literature review by Bulanda et al. (13% to 49%) [15]. The authors point out that for children the most reliable clinical method to diagnose bruxism is the reporting of teeth grinding by parents or caregivers. However, others claim that most children sleep away from their parents, and parents are not always aware of their child’s bruxism [38]. This may lead to the relatively low prevalence of SB-positive children when assessed according to parental reports. 

In the present study, there were significant differences between SB-positive and SB-negative children in the parameter of mouth breathing. This coincides with Oh et al. [36], who showed that impaired nasal breathing and mouth breathing when awake or asleep are significant risk factors for probable SB in children 6–12 years old [39]. Mouth breathing carries potential negative consequences for children, such as increasing the risk of sleep-related breathing disorders [22] by almost five times. In the present study, mouth breathing increased the risk of SB in children by almost three times. When mouth breathing is indicated during the child’s initial anamnesis, it should be further referred to and explored. 

Several studies looked into the interface between dentistry and respiratory disorders in children and suggested a positive correlation between mouth breathing and changes in the stomatognathic system [22,39]. Mouth breathing carries potential negative consequences in children, such as sleep-related breathing disorders, and should not be referred to lightly. Although present findings did not indicate that respiratory disorders increase the risk of SB, significant differences in the prevalence of respiratory disorders were detected between SB-positive and SB-negative children (33% among SB-positive children compared to only 15% of the SB-negative group). This suggests that a connection might exist between respiratory disorders and SB. 

Serra-Negra et al. [40] showed that snoring children have a higher probability of SB. Children who snore or have nightmares are more likely to develop bruxism while sleeping [9]. Thus, certain sleep quality attributes can serve as indicators for the early diagnosis of bruxism. The present results are consistent with the findings that suggested an association between SB and snoring and between snoring and respiratory disorders during sleep [41,42]. The occurrence of snoring and SB may seem like a paradox [36,38]. However, cyclic sleep characteristics associated with the hypothesis that bruxism is the result of a central mechanism involved in the maintenance of upper airway patency and esophageal lubrication during sleep may explain snoring and SB in the same child, although at a different time [43,44,45].

Some studies showed an association between sleep bruxism [46] and the habits of finger sucking, the use of pacifiers and/or bottles. Present results support this notion (children who used a pacifier and or sucked their finger showed a higher prevalence of SB than children who did not perform these habits). Lamera Lins et al. [39] suggest that oral habits such as finger sucking, pacifier use, or nail biting could be considered coping mechanisms used to unleash stress, which can also be the case for SB. 

Children and adults differ in many clinical features. In the present study, three factors emerged as possible predictors of possible SB in children—mouth breathing and ENT problems (increasing the odds by approximately three times) and use of a pacifier or finger sucking (increasing the odds by approximately two times). The prevalence of ENT problems such as ear infections in children is higher than among adults [47]. It was found that in children habitual snoring and habitual mouth breathing are highly associated with more frequent bouts of rhino sinusitis, ear infections, and antibiotic use [48]. While in adults the Eustachian tubes are more vertical and wider, allowing quick fluid drainage, in young children they are short and horizontal, leading to difficulty in drainage from the middle ear. The present findings show that children with periodical fluid drainage from the median ear, or those who have undergone ear tube insertion, are at almost three times higher risk of presenting SB behavior, which suggests that they indeed might have been suffering from otitis media more often than children who do not perform SB. 

Usually, the Eustachian tubes are collapsed at rest and open during activities such as chewing, swallowing, or yawning. The muscles that contract to help regulate the tubal opening are the medial and lateral pterygoids [47,48,49,50,51,52]. Medial and lateral pterygoids are also the muscles that are involved in mandible movements and in SB [53]. It is therefore plausible that in children, SB acts as a protective behavior in order to adjust the pressure in the Eustachian tubes, prevent Eustachian tube dysfunction, and prevent the development of inflammatory conditions of the inner ear. Otitis media in early childhood showed a detectable impact on language development in later childhood that was not accounted for by sociodemographic factors [54]. Thus, SB behavior in early childhood may be a physiological way to prevent such deleterious effects.

SB is mostly considered a behavior carrying various clinical consequences, mostly negative ones. Previous studies suggested a relationship between SB and TMD in children. SB can cause tooth wear, masticatory muscle pain, TMD pain, limited mouth opening, ear pain, and headaches [30]. Balasubramaniam et al. [55] summarized the negative consequences possibly associated with SB, such as occlusal and incisal wear, tooth fracture, TMJ discomfort or pain, and even symptoms such as a reduction in salivary flow and/or xerostomia. However, the authors qualify that some of these conditions are commonly associated with SB by clinicians, based on clinical experience, with little evidence of cause-and-effect relationships. 

The present study demonstrates that SB is often secondary to other underlying medical conditions, which can be important for the developing child’s oro-facial growth, development, and quality of life. Bruxism, snoring, and mouth breathing are common symptoms among children which are often disregarded by parents [56]. It is important to develop multidimensional evaluation tools for SB in children that are not defined in terms of the simple dichotomy of ‘present versus absent’. Regretfully, a standard tool for the assessment of bruxism (STAB), such as the one recently published for use in adults [57], does not yet refer to children. Further studies to validate parental reports through instruments relying on artificial intelligence are necessary. Applications that record snoring and bruxing sounds during sleep over a period of time may serve as helpful tools to assess SB and comorbid conditions. 

SB, both in adults and children, is still an enigma. It is today generally accepted that it is not a movement disorder or a sleep disorder in otherwise healthy individuals [12]. When the primary dentition is involved, attrition of the hard tooth tissues might not be a clinical problem, and SB behavior might be rather beneficial in preventing other pathological conditions such as otitis media. 

Further research is important to define more accurate modes of assessing SB in young children as well as its clinical consequences. While polysomnography, including EMG activity of the masticatory muscles, preferably with audio and video recordings, is the gold standard for SB diagnosis [2], other diagnostic modes are being developed and tested. In recent years, some interesting mobile devices have been introduced to facilitate data collection. These include handheld devices that receive combined EMG and electrocardiographic traces, which showed increased accuracy compared to only EMG-based devices and present a promising diagnostic approach for SB diagnosis [58,59].

Limitations: No study is without limitations. In the present study, possible SB definition was based on parental reports and not on instrumental evaluation, which is the gold standard in SB diagnosis. Moreover, current evidence suggests that bruxism should not be evaluated as a dichotomy of “positive vs. negative”. Regretfully, the current lack of verified criteria for the diagnosis of SB in children contributes to discrepancies in the literature. This study was planned as an exploratory study to evaluate the role of anamnestic parameters as an initial signal for the presence of possible SB in young children. It is, however, advisable that further studies, with a larger number of SB-positive children and a more accurate SB assessment mode, are carried out to address this issue.

## 5. Conclusions

History of ENT disorders (periodical fluid drainage from the median ear or insertion of ear tubes), mouth breathing, finger sucking or use of pacifiers, significantly increase the odds of SB in children. Clinicians should look for clinical signs of possible SB in children whose anamnesis includes one or more of the above-mentioned parameters. 

## Figures and Tables

**Table 1:** Comparisons between SB-positive and SB-negative groups.

	Group	SB Negative (%) (437) *	SB Positive (%) (84)	Total	*p* **
Anamnestic Variables					
**ENT (fluid drain/ear tubes)**					
Negative		86.1% (404)	13.9% (65)	100% (469)	**0.000**
Positive		63.5% (33)	36.5% (19)	100% (52)	
Tonsillectomy					
Negative		84.4% (421)	15.6% (78)	100% (499)	NS
Positive		72.7% (16)	27.3% (6)	100% (22)	
**Respiratory disorders**					
Negative		84.8% (419)	15.2% (75)	100% (494)	**0.026**
Positive		66.7% (18)	33.3% (9)	100% (27)	
Speech Disorders					
Negative		83.8% (409)	16.2% (79)	100% (488)	NS
Positive		84.8% (28)	15.2% (5)	100% (33)	
Night drinking					
Negative		84.2% (335)	15.8% (63)	100% (398)	NS
Positive		82.9% (102)	17.1% (21)	100% (123)	
Nail biting					
Negative		84% (362)	16% (69)	100% (431)	NS
Positive		83.3% (75)	16.7% (15)	100% (90)	
**Pacifier and/or finger sucking**					
Negative		88.4% (274)	11.6% (36)	100% (310)	**0.001**
Positive		77.3% (163)	22.7% (48)	100% (211)	
**Mouth breathing**					
Negative		88.2% (358)	11.85 (48)	100% (406)	
Positive		68.7% (79)	31.3% (36)	100% (115)	**0.000**
Tongue thrust					
Negative		84.1% (408)	15.9% (77)	100% (485)	NS
Positive		80.6% (29)	19.4% (7)	100% (36)	
**Snoring**					
Negative		85.9% (396)	14.1% (65)	100% (461)	**0.001**
Positive		68.3% (41)	31.7% (19)	100% (60)	
Ritalin					
Negative		83.9% (423)	16.1% (81)	100% (504)	NS
Positive		82.4% (14)	17.6% (3)	100% (17)	

* Percent of subjects classified to each of the groups (SB negative versus SB positive) per variable; in parenthesis—number of subjects. ** Chi-square, significant results in **bold**.

**Table 2:** Stepwise logistic regression.

Variables	B	S.E.	Wald	df	Sig.	ODDS	95% C.I. for ODDS	
							Lower	Upper
Mouth breathing	1.068	0.261	16.740	1	0.000	2.908	1.744	4.850
ENT *	1.072	0.331	10.478	1	0.001	2.921	1.526	5.590
Pacifier/finger sucking	0.678	0.251	7.325	1	0.007	1.971	1.206	3.222

* Ear, Nose, and Throat disorders—periodic fluid drain from the middle ear or application of ear tubes.

## Data Availability

The datasets generated and/or analyzed during the current study are available from the corresponding author upon reasonable request.
